# Comparison of repetitive transcranial magnetic stimulation and intermittent theta burst stimulation efficacy in treating post-stroke dysphagia: a prospective, single-blind, randomized controlled study

**DOI:** 10.3389/fneur.2025.1650216

**Published:** 2025-10-10

**Authors:** Fang Li, Jinling Cheng, Yanying Zhu, Yang Peng, Zicai Liu

**Affiliations:** ^1^Department of Rehabilitation Medicine, Yuebei People’s Hospital, Shaoguan, Guangdong, China; ^2^Department of Rehabilitation Medicine, Shaoguan First People’s Hospital, Shaoguan, Guangdong, China

**Keywords:** dysphagia, repetitive transcranial magnetic stimulation, stroke, intermittent theta burst stimulation, randomized controlled study

## Abstract

**Objective:**

To compare the efficacy of repetitive transcranial magnetic stimulation (rTMS) and intermittent theta burst stimulation (iTBS) applied to the motor cortex representation of the mylohyoid muscle in treating post-stroke dysphagia.

**Methods:**

Ninety-two patients with post-stroke dysphagia (July 2022–May 2023) were randomized into three groups: rTMS (*n* = 31), iTBS (*n* = 30), and control (*n* = 31). The rTMS and iTBS groups received respective stimulations plus routine rehabilitation; the control group received routine rehabilitation alone. Swallowing function was assessed pre- and post-intervention using the Penetration-Aspiration Scale (PAS) and Dysphagia Disability Index (DD).

**Results:**

After 2 weeks, all groups showed significant swallowing improvement (*p* < 0.001). Both rTMS and iTBS groups demonstrated greater improvement in PAS and DD scores versus controls (*p* < 0.001). No significant difference emerged between rTMS and iTBS efficacy (*p* > 0.05).

**Conclusion:**

rTMS and iTBS equivalently improve post-stroke dysphagia. iTBS achieves comparable outcomes with shorter treatment duration, supporting its clinical adoption.

**Clinical trial registration:**

Identifier ChiCTR2200058246, https://www.chictr.org.cn/.

## 1 Introduction

Post-stroke dysphagia affects 45–78% of survivors (1), increasing risks of aspiration pneumonia and mortality (2). Neurostimulation techniques like rTMS modulate cortical excitability to promote swallowing recovery (3). While high-frequency rTMS applied to the unaffected hemisphere improves dysphagia by rebalancing interhemispheric inhibition (4), its 20–30 min/session protocol challenges clinical feasibility. Intermittent theta burst stimulation (iTBS) (5), transcranial direct current stimulation (tDCS) (6), neuromuscular electrical stimulation (NMES) (7), pharyngeal electrical stimulation (PES) (8), and traditional swallowing rehabilitation are common technology for dysphagia. The conventional methods include swallowing muscle training, health education, and dietary modification (9). Transcranial magnetic stimulation (TMS) modalities such as rTMS (10) and iTBS utilize magnetic fields to generate cortical currents that modulate neuronal excitability (11).

rTMS is an FDA-approved non-invasive neuromodulation technique (12). rTMS generates induced currents to cause changes in the excitability of the cortex at the corresponding site through a continuous repetitive stimulation pattern (13). Many studies have shown that rTMS can effectively improve dysphagia in stroke patients, without adverse effects. In a meta-analysis in 2017, Liao et al. (14) pointed out that high-frequency may be more effective than low-frequency TMS, and simultaneous stimulation of the healthy side or bilateral hemispheres has a significant effect. iTBS is a plexiform rhythmic stimulation added to the basis of rTMS. The effects of iTBS have been demonstrated for the human pharyngeal motor system (15), which has the characteristics of a more stable and longer-lasting alteration of cortical excitability in comparison with rTMS, and it can be used in a shorter time and with lower intensity to achieve a better effect (16). Currently, iTBS is also gradually used to improve the swallowing function of post-stroke patients, such as Rao et al. (17) by bilateral iTBS stimulation of the cerebellar hemispheres can effectively improve dysphagia, no adverse reactions occurred. Suh et al. (18) 5-min iTBS treatment on the affected side of the first post-stroke residual swallowing disorders, compared with the sham iTBS group, the patients’ PAS and FDS had a greater improvement. No adverse reactions occurred.

Notably, neurostimulation targeting the pharyngeal motor cortex has demonstrated efficacy in post-stroke dysphagia rehabilitation. Michou et al. (19) first established that high-frequency rTMS applied to the unlesioned pharyngeal cortex significantly improves swallowing function by rebalancing interhemispheric inhibition. Both high-frequency rTMS and iTBS can promote the excitation of neurons at the corresponding stimulation sites in the cerebral cortex. To further compare the therapeutic efficacy of rTMS with that of iTBS. Yu-Lei et al. (20) conducted a randomized controlled trial for the first time, and stimulation of the supraglottic muscles using high-frequency (10 Hz) rTMS or iTBS significantly improved dysphagia, with no efficacy difference between modalities. There was no significant difference in efficacy, while the duration of action was shorter in the iTBS group. However, there was no control group in this study, the number of stimulation pulses was inconsistent between the two stimulation modalities, and some assessments were incomplete after two weeks of intervention.

To further compare the efficacy of rTMS and iTBS, the present study employed three interventions: 5 Hz rTMS, iTBS, and conventional treatment. These were applied to the representative area of the motor cortex of the mylohyoid muscle to evaluate their effectiveness. Additionally, the stimulation site was alternated to the healthy side to assess the significance of the efficacy, providing a basis for the follow-up of clinical rehabilitation treatment. This study will begin in 2022 with patient recruitment and treatment. Our study extends this evidence by rigorously comparing rTMS and iTBS protocols matched for pulse number—a critical design consideration given the dose-dependent effects of iTBS on cortical excitability (21). Unlike Yu-Lei et al. (20), our study: (1) included a control group, (2) matched pulse counts (1,200 pulses) between rTMS and iTBS protocols to isolate stimulation-pattern effects, and (3) standardized post-intervention assessments.

## 2 Methods

### 2.1 Participants

**Figure 1:**
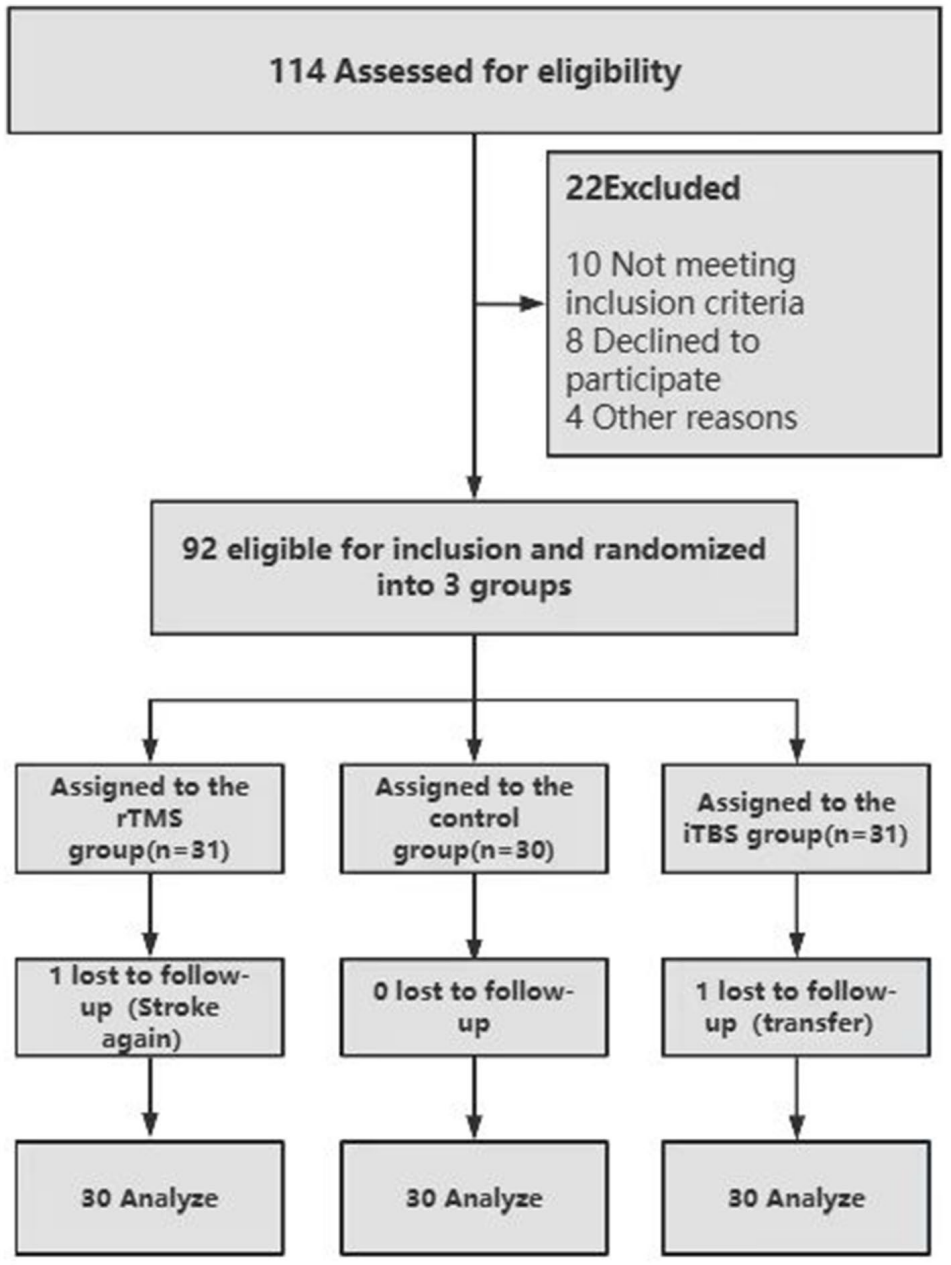
CONSORT diagram.

This study was conducted on patients with post-stroke dysphagia. Ninety-two patients with post-stroke dysphagia who were admitted to Yuebei People’s Hospital in Shaoguan City, Guangdong Province, from July 2022 to May 2023 were selected. The 92 patients were evaluated based on the following inclusion criteria: stroke diagnosed via computed tomography (CT) or magnetic resonance imaging (MRI); dysphagia diagnosed through fiberoptic endoscopic examination of swallowing; and patients who were emotionally stable and capable of cooperating to complete the trial. Exclusion criteria: concomitant other neurological disorders such as epilepsy, Parkinson’s disease, or severe disorders of consciousness; contraindications to electrical or magnetic stimulation. The 92 patients were divided into 3 groups according to the intervention: the rTMS group (31 patients), the iTBS group (30 patients), and the control group (31 patients). The specific inclusion flow chart is in Figure 1. Finally, one patient in the rTMS group was lost due to another stroke, one patient in the control group was lost due to transfer to another hospital, and the remaining 90 patients completed the 2-week evaluation and treatment. Informed consent was obtained from all participants, and the study was approved by the Ethics Committee of Yuebei People’s Hospital. The ethical approval number is KY-2022-115. This study is based on the CONSORT stated reporting specification and also follows the requirements of the Declaration of Helsinki, clinical registration number: ChiCTR2200058246.

### 2.2 Interventions

This study was a single-blind, randomized controlled trial. Assessors remained blinded to group allocation throughout data collection and analysis. The rTMS group received repetitive transcranial magnetic stimulation (rTMS) stimulation in addition to conventional swallowing training, while the iTBS group was given transcranial magnetic stimulation in the intermittent theta burst stimulation (iTBS) mode along with conventional swallowing training. The control group only received conventional swallowing training. The stimulation site was the representative area of the motor cortex corresponding to the mylohyoid muscle on the healthy side (if the patient had experienced bilateral strokes, the site with the lesser degree of stroke was selected for stimulation), and the treatment was conducted by a professional while holding the coil at a 90° angle to the scalp. Routine swallowing training encompassed breathing exercises, sensory and motor training of the oropharynx, and the Mendelsohn maneuver. All patients completed the training 1 time (t)/day (d), 5 times a week for 2 weeks. rTMS group received a total of 10 sessions of rTMS stimulation with a single treatment time of 20 min, stimulation frequency of 5 Hz, stimulation time of 2 s, inter-stimulation time of 10 s, and repetition period of 100, for a total of 1,200 pulses. iTBS group received a total of 10 sessions of iTBS stimulation with a single treatment time of 6 min, intra-cluster frequency 50 Hz, intra-cluster count 3, intra-cluster stimulation time 0.06 s, inter-cluster frequency 5 Hz, inter-cluster count 10, stimulation time 2 s, intermittent time 8 s, repetition number 40, total 1,200 pulses. In the control group, routine swallowing training was performed 1×/day, 30 min/session, 5 t/week for a total of 2 weeks. The equipment used was a MagPro CCY-I transcranial magnetic stimulator (Wuhan Iridium Medical Equipment New Technology Co., Ltd., China), and the stimulation coil was an “8” coil with a peripheral diameter of 9 cm. The mylohyoid motor cortex representation was localized using the international 10–20 EEG system, while neuronavigation is the gold standard, 10–20 systems have been validated to have acceptable effectiveness in resource-limited environments. Based on probabilistic mapping of corticopharyngeal networks (22), the stimulation target was set at 3 cm anterior and 5 cm lateral to the vertex (Cz). This site corresponds to the lateral precentral gyrus, where maximal motor evoked potentials in mylohyoid muscles are elicited, as validated by transcranial cortex-to-scalp projection modeling (23).

Resting motor threshold (RMT) was determined before each session by delivering single pulses over the target site. RMT was defined as the minimum intensity required to elicit motor-evoked potentials ≥50 μV in the contralateral mylohyoid muscle in 5 of 10 trials. All stimulations were delivered at 90% RMT. Although 600 pulses are a common iTBS dose, we matched total pulses (1,200) across rTMS and iTBS groups to isolate the effect of stimulation pattern rather than dose. This approach aligns with established protocols for comparative neuromodulation studies (24) and accounts for evidence that extended iTBS protocols may shift from facilitatory to inhibitory effects. Stimulation of the unlesioned hemisphere was selected based on evidence that disinhibition of the intact cortex promotes functional recovery in unilateral stroke (25, 26). For bilateral strokes, the hemisphere with higher Fugl-Meyer scores was designated as “less lesioned,” consistent with established criteria for identifying salvageable motor networks (27).

### 2.3 Outcome measure

Swallowing assessments were conducted by two blinded speech-language pathologists (FL and YZ) with >5 years’ experience. PAS scores were derived from flexible endoscopic evaluation of swallowing (FEES) performed within 48 h pre- and post-intervention. Participants swallowed 5-mL thin liquid barium boluses in lateral view, with PAS rated frame-by-frame. The Dysphagia Disability Index (DD) was assessed via clinical examination of oral/pharyngeal function.

#### 2.3.1 Rosenbek Penetration/Aspiration Scale

The scale assesses swallowing function during the pharyngeal phase of the patient, and the specific procedure is to score the degree of intrusion of the ingested food mass into the patient’s airway and the ability to clear the foreign body by observing it under FEES. Grade 1: food does not enter the airway with no leakage of aspiration; grades 2–4: food enters the airway with varying degrees of leakage; grades 5–8: food enters the airway that adheres to the vocal cords in severe cases reaching below the vocal cords and aspiration occurs. The higher the level of this score, the more severe the swallowing disorder (28).

#### 2.3.2 Dysphagia Disability Index

DD-I has no clinical signs or symptoms of dysphagia.
DD-II is mild dysphagia that is not detected by the patient.
DD-III is a patient who complains of dysphagia but can eat through the mouth.
DD-IV is when the patient has significant dysphagia and may not eat through the mouth.

The higher the level of this score, the more severe the dysphagia (29).

### 2.4 Statistical analysis

The analysis encompassed measures such as the median (interquartile range, IQR) for continuous variables, and frequencies along with percentages for categorical variables. Group comparisons for continuous variables were conducted using the Wilcoxon rank-sum test or the Kruskal–Wallis test. For the comparison between groups of categorical data, the Fisher exact test was employed when expected frequencies <were less than 5; otherwise, the chi-squared test was utilized.

The study design determines the method of controlling the balance of covariates between groups. This study is a classical clinical trial study design, and the demographic or clinical characteristics of the groups have already been balanced by randomized grouping, we used the double-difference method to evaluate the efficacy of the different groups. Subgroup analyses were used to look at different subgroups (gender, stroke type, and lesion side) and whether there is any difference in efficacy, the least squares mean and its confidence interval based on the ANOVA model and the *p*-value showing the interaction can help to determine the difference between different groups, so we selected the PAS as an outcome indicator to draw the forest plot for subgroup analysis.

In our study, all statistical analyses were performed using the R software (version 4.2.2).

## 3 Results

A total of 114 patients were evaluated in this study, out of which 10 patients were excluded as they did not fulfill the inclusion criteria, 8 patients refused to join the study, and 4 patients did not participate for other reasons as well, finally, 92 patients were included. After a period of intervention, rTMS lost one patient and the control group lost one patient, a total of 90 patients completed the study with no significant adverse effects. The baseline characteristics of participants across the three groups are revealed in Table 1. The median ages were similar across groups, with the rTMS group at 65 years, the iTBS group at 70 years, and the Control group at 69 years; differences were not statistically significant (*p* = 0.928). The disease duration showed no notable variation between groups (*p* = 0.779). There was no significant difference in the gender distribution ratio among the three groups (*p* = 0.350). For lesion side characteristics, distribution values show non-significant differences (*p* = 0.620). Regarding stroke type, the configuration was predominantly statistically non-significant (*p* = 0.298). Measures such as the water-swallowing test (WST) and the standardized swallowing assessment (SSA) were similar across all groups (with *p*-values of 0.327 and 0.858, respectively), indicating baseline homogeneity in these parameters. Overall, the groups exhibited comparability across most baseline characteristics.

**Table 1:** Patient demographics and baseline characteristics.

Characteristic	Group		
	rTMS, *N* = 30^a^	iTBS, *N* = 30^a^	Control, *N* = 30^a^
Age	65 (56, 79)	70 (59, 74)	69 (61, 75)
Disease duration	34 (22, 59)	32 (13, 68)	34 (18, 45)
Sex			
Male	17 (56.7%)	22 (73.3%)	21 (70.0%)
Female	13 (43.3%)	8 (26.7%)	9 (30.0%)
Lesion side			
Right hemisphere	11 (36.7%)	10 (33.3%)	12 (40.0%)
Left hemisphere	6 (20.0%)	10 (33.3%)	10 (33.3%)
Bilateral cerebral hemispheres	13 (43.3%)	10 (33.3%)	8 (26.7%)
Stroke type			
Hemorrhage	9 (30.0%)	9 (30.0%)	14 (46.7%)
Infarction	21 (70.0%)	21 (70.0%)	16 (53.3%)
WST	4.00 (4.00, 5.00)	4.00 (4.00, 5.00)	4.00 (4.00, 5.00)
SSA	25.5 (22.0, 28.0)	24.0 (20.3, 27.8)	25.0 (22.0, 27.8)

rTMS, repetitive transcranial magnetic stimulation; iTBS, intermittent theta burst stimulation; WST, water-swallowing test; SSA, standardized swallowing assessment.

a Median (IQR); *n* (%).

### 3.1 Swallowing function

After 2 weeks of treatment, the DD and PAS scores for all three groups were lower than they were before the treatment, and these differences were statistically significant (*p* < 0.05). rTMS group before and after treatment DD (LS mean = −0.77; 95% CI: −0.97 to 0.57); iTBS group before and after treatment DD (LS mean = − 0.69; 95% CI: −0.89 to 0.49); pre- and post-treatment DD in the control group (LS mean = −0.13 to 0.07). Pre- and post-treatment PAS in the rTMS group (LS mean = −0.2.05; 95% CI: −2.34 to 1.76); pre- and post-treatment PAS in the iTBS group (LS mean = −2.17; 95% CI: −2.46 to 1.88); pre- and post-treatment PAS in the control group (LS mean = −0.48; 95% CI: −0.77 to 0.19). rTMS and iTBS groups had lower DD and PAS scores than the control group (*p* < 0.05); the difference in PAS and DD scores before and after the intervention was not statistically significant in the rTMS group compared to the iTBS group (*p* > 0.05). rTMS VS iTBS on DD scores (*p* = 0.847); rTMS VS iTBS on PAS scores (*p* = 0.83). Detailed results are shown in Tables 2–4.

**Table 2:** DD Comparison of the three subgroups before and after treatment.

Group	Baseline	Post-treatment	Change from baseline	
	Mean (SD)	Mean (SD)	Mean (SD)	LS mean (95% CI)^a^
rTMS	3.7 (0.48)	2.9 (0.88)	−0.8 (0.68)	−0.77 (−0.97, −0.57)
iTBS	3.6 (0.50)	2.9 (0.88)	−0.7 (0.60)	−0.69 (−0.89, −0.49)
Control	3.6 (0.61)	3.5 (0.73)	−0.1 (0.35)	−0.13 (−0.33, 0.07)

iTBS, intermittent theta burst stimulation; rTMS, repetitive transcranial magnetic stimulation; ANCOVA, analysis of covariance; CI, confidence interval; LS, least squares; SD, standard deviation.

a Based on an ANCOVA model after adjusting baseline value.

**Table 3:** PAS comparison of the three subgroups before and after treatment.

Group	Baseline	Post-treatment	Change from baseline	
	Mean (SD)	Mean (SD)	Mean (SD)	LS mean (95% CI)^a^
rTMS	5.3 (1.60)	3.2 (1.54)	−2.1 (0.83)	−2.05 (−2.34, −1.76)
iTBS	5.1 (1.67)	2.9 (1.74)	−2.2 (0.91)	−2.17 (−2.46, −1.88)
Control	5.0 (1.67)	4.6 (1.68)	−0.5 (0.68)	−0.48 (−0.77, −0.19)

iTBS, intermittent theta burst stimulation; rTMS, repetitive transcranial magnetic stimulation; ANCOVA, analysis of covariance; CI, confidence interval; SD, standard deviation.

a Based on an ANCOVA model after adjusting baseline value.

**Table 4:** Three subgroups of two-by-two comparisons.

Pairwise comparison	DD		PAS	
	Difference in LS mean (95% CI)^a^	*p*-value	Difference in LS mean (95% CI)^a^	*p*-value
iTBS—rTMS	0.08 (−0.20, 0.36)	0.847	−0.12 (−0.52, 0.28)	0.830
Control—rTMS	0.64 (0.36, 0.92)	<0.001	1.57 (1.17, 1.98)	<0.001
Control—iTBS	0.56 (0.28, 0.84)	<0.001	1.69 (1.29, 2.09)	<0.001

iTBS, intermittent theta burst stimulation; rTMS, repetitive transcranial magnetic stimulation; ANCOVA, analysis of covariance; CI, confidence interval; LS, least squares.

a Based on an ANCOVA model after adjusting baseline value.

### 3.2 Subgroup analyses

**Figure 2:**
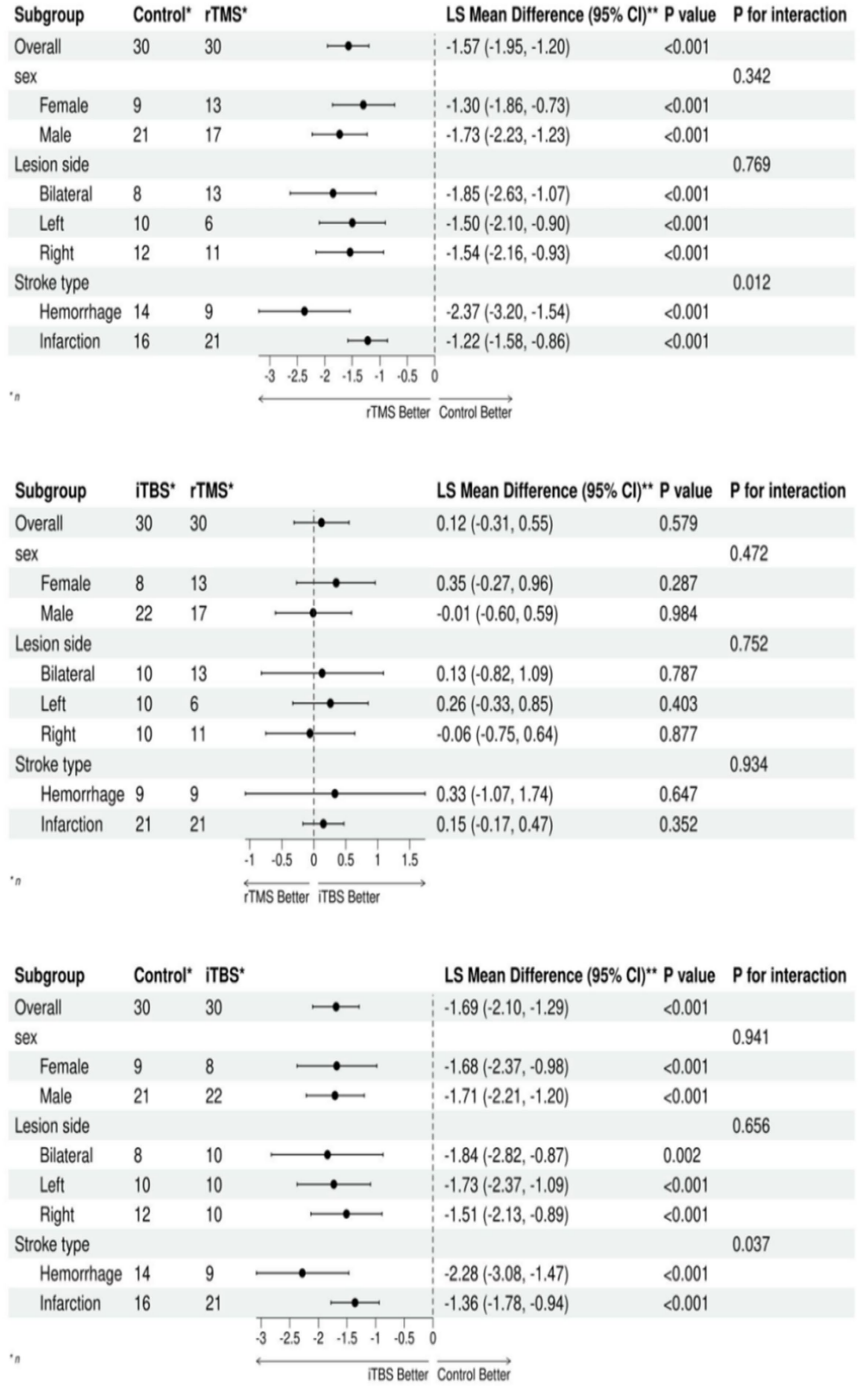
Forest plot of the subgroup analysis based on the PAS.

Upon completion of the treatment, subgroup analyses of PAS, the primary outcome measure, were conducted to evaluate the impact of gender, lesion site, and lesion type on treatment effectiveness. The findings indicated no significant variation in treatment effectiveness between genders and lesion sites (*p* > 0.05). Both the rTMS and iTBS groups demonstrated an advantage over the control group, with the cerebral hemorrhage population experiencing greater efficacy compared to those with cerebral infarction (*p* < 0.05). For further details, refer to Figure 2.

### 3.3 Safety analysis

No serious adverse reactions were found in any of the three groups.

## 4 Discussion

The results of the study showed that both conventional swallowing training and the addition of 5 Hz rTMS or iTBS to conventional training to stimulate the motor cortical representative area of the healthy mylohyoid muscle were effective in improving swallowing disorders in post-stroke patients, and the efficacy of the treatment was not related to age, gender, and damaged site. Comparing the conventional training groups, the rTMS, and iTBS groups had better therapeutic effects, indicating that 5 Hz rTMS and iTBS combined with conventional training are more effective in promoting the recovery of swallowing function in post-stroke patients. In this study, both rTMS and iTBS groups received 10 sessions of magnetic stimulation, and the number of pulses (1,200) was the same in both groups. There was no significant difference in the PAS scores and DD scores between the iTBS group that received the intervention for 6 min and the rTMS group that received it for 20 min. This is similar to Yu-Lei et al. (20) who utilized 10 Hz rTMS and iTBS for dysphagia after stroke, PAS scores of the two groups PAS scores were reduced more significantly after 2 weeks of treatment in this study.

Swallowing is a complex process that requires the coordinated work of at least 25 muscles to complete (30). Among them, the supraglottic muscle (including the mylohyoid muscle) is an important part of the swallowing reflex, which can contract and lift the hyoid bone to push and squeeze the food mass into the pharynx, which is innervated by the bilateral motor cortical areas of the brain and there is the asymmetry in the innervation (31). Post-stroke swallowing dysfunction is believed to be associated with an imbalance in interhemispheric inhibitory mechanisms (26). Neurophysiologically, this is characterized by a reduction in excitability of the affected hemisphere and an abnormal elevation in excitability of the healthy hemisphere. The heightened excitability of the healthy hemisphere suppresses the affected hemisphere, leading to decreased (32) excitability. Neuroplasticity is often used clinically to improve dysphagia by modulating cortical excitability (33). rTMS and iTBS are commonly used neuromodulation techniques for the treatment of post-stroke dysphagia (34). rTMS and iTBS may have a mechanism of action that involves the joint enhancement of swallowing by stimulating the innervation of certain overlapping cortical areas in the oral cavity and pharynx (35, 36). The sites of action of rTMS and iTBS have not yet been clarified. Tarameshlu et al. (37) noted in a randomized controlled trial that 1 Hz rTMS applied to the cortical areas of the healthy subglottic muscle group could inhibit its excitability and improve the swallowing function of the patients; Park et al. (38) administered 5 Hz rTMS to the motor cortex of the pharynx on the affected side of patients with dysphagia after stroke; and the patients were treated for 2 weeks after the treatment (39). In this study, 5 Hz rTMS and iTBS were applied to the motor cortex of the mylohyoid muscle to improve excitability, and both results could effectively improve swallowing disorders. This suggests that rTMS acting on either the affected or healthy side can improve swallowing function in patients after stroke.

In conclusion, both repetitive transcranial magnetic stimulation (rTMS) and intermittent theta burst stimulation (iTBS) can enhance swallowing function. Their efficacies are not significantly different; however, iTBS has a shorter effect time, which greatly improves the acceptability of magnetic stimulation and reduces the dropout rate during treatment. There is no significant difference between rTMS and iTBS in improving swallowing disorders post-stroke, which may be attributed to various factors, including the number of intervention pulses, intervention frequency, intervention intensity, and intervention site. Further studies are required to ascertain the optimal parameters. For instance, it may be beneficial to design multiple groups using orthogonal experiments to compare the differences in efficacy.

The strengths of this study are that it further expanded the previously unprecedented stimulation site (motor cortical area of the hyus muscle), and then used the intervention with rTMS and iTBS with the same pulse number in addition to the basic intervention period, increasing the comparability of the two stimuli. The limitations of this study include an insufficient comprehensive understanding of basic information of patients, such as BMI, hypertension, hyperglycemia, etc.; subjective judgment on the stimulation site for patients with bilateral stroke; insufficient comprehensive monitoring data and objective indicators (such as near-infrared, magnetic resonance imaging, pharyngeal pressure measurement or pharyngeal electromyography, etc.). Finally, the selection of outcome indicators was based on relevant literature, and we do not exclude the possibility that there may be certain risks affecting the results.

## 5 Conclusion

Both 5 Hz rTMS and iTBS can improve swallowing disorders in stroke patients. The iTBS can achieve a similar efficacy to the rTMS in a shorter time, which can be promoted in clinical practice later.

## Data Availability

The datasets presented in this study can be found in online repositories. The names of the repository/repositories and accession number(s) can be found in the article/supplementary material.
